# Identifying knowledge gaps in reproductive health management for women with multiple sclerosis and healthcare professionals: a scoping review

**DOI:** 10.3389/fneur.2026.1769826

**Published:** 2026-03-20

**Authors:** Claudia De Santis, Riley Bove, Dina Jacobs, Jennifer McDonell, Francesco Nonino, Nick Rijke, Deanna Saylor, Shanthi Viswanathan, Elisa Baldin

**Affiliations:** 1 Epidemiology and Statistics Unit, IRCCS, Istituto delle Scienze Neurologiche di Bologna, Bologna, Italy; 2 UCSF Weill Institute for Neurosciences, University of California San Francisco, San Francisco, CA, United States; 3 Department of Neurology, Perelman School of Medicine, University of Pennsylvania, Philadelphia, PA, United States; 4 MS Canada, Toronto, ON, Canada; 5 Multiple Sclerosis International Federation, London, United Kingdom; 6 Chapell Hill School of Medicine, University of North Carolina, University Teaching Hospital, Lusaka, Zambia; 7 Department of Neurology, Kuala Lumpur Hospital, Tunku Abdul Rahman Neuroscience institute, Kuala Lumpur, Malaysia

**Keywords:** information, knowledge gap, multiple sclerosis, reproductive health, scoping review, women

## Abstract

Research indicates that pregnancy does not negatively impact the long-term progression of multiple sclerosis (MS); however, women with MS (WwMS) often do not have access to consistent and reliable information on reproductive health, particularly in settings with limited resources. This scoping review aims at providing an overview of the current evidence on knowledge gaps and information needs on reproductive health by WwMS in childbearing age globally. The review was conducted in May 2025 by searching three databases for peer-reviewed studies involving adult women with a diagnosis of MS and/or addressing their knowledge on reproductive health. Studies on professionals engaged in MS care as providers of information about reproductive health were also included. Data were categorized according to 13 key reproductive health indicators. Finally, of 545 records screened, 29 studies met the inclusion criteria. Most studies included only WwMS (59%) and were based in high-income countries (72%). Results are presented in an interactive scoping map showing the distribution of key health indicators across study characteristics such as geographic area, study design, and methods. This review highlights gaps in studies targeting the experience of WwMS in managing reproductive health in low- and middle-income countries.

**Systematic review registration:**

The scoping review was registered on Zenodo at the following link: https://zenodo.org/records/13866567.

## Introduction

Multiple sclerosis (MS) is a chronic autoimmune condition affecting approximately 3 million people worldwide, with an increasing prevalence observed across most regions globally (1). Women are disproportionately affected by MS compared to men, with a ratio of 2–3 to 1. MS is commonly diagnosed between the ages of 20 and 50, which are the prime reproductive years for women. Evidence suggests that pregnancy does not adversely affect the long-term course of MS (2), with MS not significantly affecting pregnancy outcomes in women (3). However, many disease-modifying therapies (DMTs) commonly used in MS care are contraindicated during pregnancy and breastfeeding (4–6). Consequently, women with MS (WwMS), as well neurologists and other health professionals engaged in MS care, face unique challenges in shared decision-making on reproductive health issues. Several studies investigated the role of neurologists as key actors in family planning, reporting significant variability in their approach, along with a lack of dedicated resources to provide information and support for family planning decisions among WwMS (7, 8). Furthermore, qualitative research indicates that a collaborative decision-making process between people with MS and healthcare providers can help patients get better information about their reproductive health (8, 9). Global MS prevalence varies across regions, with lower-income countries potentially experiencing underdiagnosis due to limited healthcare resources (1). Studies assessing reproductive health issues in MS are mostly based in high-income countries, resulting in a notable gap in the understanding of the experiences of WwMS regarding breastfeeding and other reproductive health issues in low- and middle-income countries (10).

A clearer understanding of the current evidence landscape and identification of areas deserving further research could inform health system decision-making on reproductive health for WwMS. By providing a systematic search of the available evidence and mapping key concepts and knowledge gaps, a scoping review allows for an exploratory overview of the field and helps guide future research when the available literature is particularly heterogeneous (11).

### Aim

The objectives of this scoping review are:

- To provide an overview of the current available evidence about knowledge gaps and information needs on reproductive health by women with multiple sclerosis at a global level.
- To provide an overview of current evidence on the role of the neurologist and MS healthcare providers in informing shared decisions regarding reproductive health management for women with multiple sclerosis of childbearing age.

## Methods

This scoping review was conducted and reported according to the JBI Manual for Evidence Synthesis methodology (11) and the PRISMA-ScR guideline (Supplementary Table 1) (12), respectively. This scoping review was guided by the PCC framework (Population, Concept, Context) as recommended (11). The population of interest was women diagnosed with multiple sclerosis. The core concept explored was the knowledge gaps and information needs surrounding reproductive health, including the perspective of healthcare professionals as an additional source of evidence. The context encompassed all aspects of reproductive health, including but not limited to pregnancy, family planning, contraception, and breastfeeding.

The protocol for this study was registered on Zenodo (13). Deviations from the original protocol can be found in the change of methodology to summarize the results. Rather than qualitative synthesis, we prioritized the development of an interactive evidence gap map to provide a more accessible and immediate synthesis of the current literature landscape. Additionally, while the PCC framework was applied throughout the study, it is explicitly presented only in the current article and not detailed in the protocol.

The results of this scoping review will inform the development of a core set of essential information that every woman with MS should know about pregnancy, breastfeeding and contraception. The whole project (Knowledge Gaps in Reproductive Health among Women with MS - KNOWwMS) was developed by an international, multidisciplinary scientific advisory group (SAG) comprising MS healthcare professionals and representatives of people with MS (Supplementary Table 2). The complete project protocol (KNOWwMS) is available on Zenodo (14).

### Search strategy

An information specialist developed a relevant search strategy to capture the available literature (Supplementary Table 3). On May 19th, 2025, the search was conducted on the PubMed, CINAHL, and Embase databases with no restrictions on time frame or language.

### Eligibility criteria

We included quantitative and qualitative primary studies of any design published on peer-reviewed journals exploring the knowledge on women reproductive health issues in the context of MS. Reviews, letters, commentaries, editorials, and abstracts were excluded.

To be included, studies had to follow the PCC framework, as outlined above.

### Study selection

References retrieved by the search were independently screened by title, abstracts and subsequently in full text by two authors (EB, CDS). Disagreements were resolved through discussion or, whenever necessary, by a third reviewer (FN). Reference lists of the included literature were manually screened in order to identify any additional eligible study.

Retrieved references were managed by means of the online software Rayyan (15).

### Data extraction

One reviewer (CDS) extracted on an ad-hoc template the following data: title, first author (year), language, population (WwMS, People with MS, MS Healthcare professionals, General population), sample size, country or region where the study was conducted, study design, methods, and study aim. A second reviewer (EB) verified the extraction.

To guide the data extraction, key concept indicators were first drafted based on the literature search and then refined with input from the SAG. Finally, 13 key indicators were selected: 8 on reproductive health from the perspective of WwMS and 5 related to clinical practice by neurologists and other professionals involved in MS reproductive health care (Table 1).

**Table 1:** List of key indicators and their sub-categories.

Key indicators	Sub-categories
WwMS reproductive health	
Access to contraception	Use of contraception
	Contraception counseling
Rate of planned vs. Unplanned pregnancies	Unplanned pregnancy
Age at first childbirth	Age at first childbirth
Access to general prenatal care	Gynecological follow-up
Completed family size for women currently aged 50–55	–
Reproductive health literacy	Knowledge gap regarding family planning
Impact of MS diagnosis on family planning and breastfeeding decisions	Family planning after MS Diagnosis
	Breastfeeding
Communication and counseling received from healthcare providers regarding reproductive health	Quality
MS professionals’ clinical practice	
Characteristics (such as frequency and quality) of the guidance provided to WwMS regarding family planning counseling	Frequency
	Quality
Knowledge gaps regarding reproductive health management for WwMS	Evidence available
	Interdisciplinary counseling
Perceived self-efficacy about providing family planning counseling	–
Clinical knowledge and use of recommendations	General recommendations
	Contraception recommendations
Adherence to regulatory labels (by FDA, EMA, and other agencies)	Adherence to regulatory labels

### Data synthesis

Data were mapped according to the key health indicators identified and the studies’ characteristics such as geographic area, income level, study design and methods. The data visualization tool EPPI Mapper (16) was employed to develop evidence gap map. Areas with the most available evidence were therefore systematically highlighted, as well as those warranting further research, offering a structured representation of the available evidence about knowledge gaps and information needs on reproductive health, using perspective of both WwMS and of MS healthcare professionals.

## Results

The initial search identified a total of 819 articles. After removing duplicates, 545 unique records were available for screening. Of these, 33 articles were considered eligible for full-text review. Following the full-text screening, 9 articles were excluded for not meeting the inclusion criteria, while 4 additional articles were identified through reference list screening. Finally, 29 articles met the inclusion criteria and were included in the data extraction.

**Figure 1:**
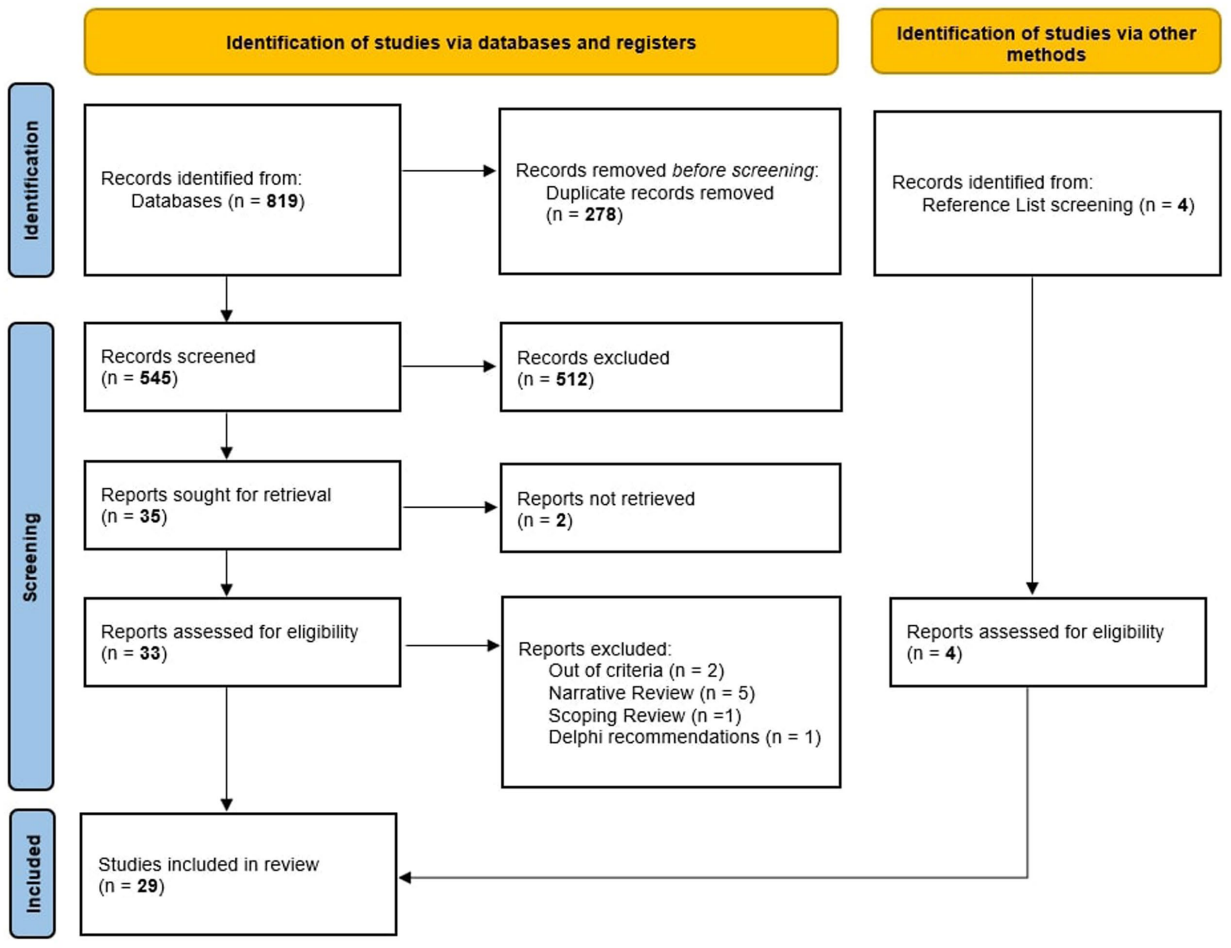
PRISMA flow diagram.

The process and results of study selection are reported in the PRISMA flow diagram (Figure 1).

### Studies characteristics

Studies ranged from 2004 to 2025. All studies were carried out in English but Vidal-de Francisco (2023), which was reported in Spanish. Seventeen studies (59%) included only WwMS. Only 4 studies included data solely from MS professionals, mostly neurologists, while 3 recorded data from both WwMS and MS professionals. Both male and female patients affected by MS were included in 5 studies.

Most studies (21/29, 72%) employed a cross-sectional design, while only two studies (7%) used a cohort approach. In terms of methodology, the majority relied on survey-based data, with fewer studies adopting qualitative (6/29, 21%) or mixed- methods (2/29, 7%).

Twenty-one studies (72%) were performed in high-income countries, 6 (21%) in upper-middle income countries and one in a low-middle income country (9). One global study did not specify all included countries, which may comprise nations with low or middle income levels (17).

Studies characteristics are described in Table 2.

**Table 2:** Studies characteristics.

Author, Year	Country	Income level	Study design	Methods	Sample	N WwMS	% WwMS	N MS p
Alanazi et al. (33)	Saudi Arabia	UMC	Cross-sectional	survey	WwMS	91	100	/
Alanazy et al. (34)	Saudi Arabia	UMC	Cross-sectional	survey	WwMS	120	100	/
Albahrani et al. (35)	Saudi Arabia	UMC	Cross-sectional	survey	WwMS	57	100	/
Albrecht et al. (36)	Germany	HIC	Cross-sectional	survey	WwMS	154	100	/
Alonso et al. (24)	Argentina	UMC	Cross-sectional	survey	WwMS	428	100	/
Alshehri (47)	Saudi Arabia	UMC	Cross - sectional	survey	WwMS	176	100	/
Bonavita et al. (37)	USA, UK, Italy, Spain, France, Germany	HIC	Cross-sectional	survey	PwMS	332	66.5	/
Borisow et al. (38)	Germany	HIC	Cross-sectional	survey	MS p	0	0	56
Carvalho et al. (39)	Portugal	HIC	Cross-sectional	survey	WwMS	100	100	/
Coyle et al. (19)	USA, Canada	HIC	Cross-sectional	survey	MS p	0	0	147
Fragkoudi et al. (7)	Australia	HIC	Qualitative study	interview	MS p	0	0	19
Fragkoudi et al. (21)	Australia	HIC	Qualitative study	interview	PwMS	31	93.5	/
Vidal-de Francisco et al. (26)	Spain	HIC	Cohort study	registry analysis	PwMS	100	86	/
Ghafoori et al. (9)	Iran	LMIC	Qualitative study	interview	WwMS	25	100	/
Kamm et al. (8)	Switzerland	HIC	Cross-sectional	survey	WwMS	271	100	/
Kelly et al. (40)	USA, Canada	HIC	Cross-sectional	survey	WwMS	280	100	/
Kosmala-Anderson Wallace (22)	UK	HIC	Qualitative study	interview	WwMS, MS p	9	100	5
Lavorgna et al. (41)	Italy	HIC	Cross-sectional	survey	PwMS	354	71	/
Payne and McPherson (42)	New Zealand	HIC	Qualitative study	interview	WwMS	9	100	/
Pebdani et al. (43)	USA	HIC	Cross-sectional	survey	WwMS	391	100	/
Peper et al. (44)	Germany	HIC	Cross-sectional	survey	WwMS	100	100	/
Perillan (48)	Spain	HIC	Cross-sectional	survey	WwMS	214	100	/
Prunty et al. (25)	Australia	HIC	Qualitative study	focus group	WwMS	20	100	/
Rasmussen et al. (27)	Denmark	HIC	Cross-sectional	survey	PwMS	488	83	/
Ross et al. (17)	USA, Canada, Europe, South Pacific	HIC and unspecified	Cross-Sectional	mixed method				
				survey	GP	1,191	83	/
				survey	GP	3,836	75	/
				survey	GP	574	81	/
				focus group	GP	51	62	5
Renaud et al. (18)	France	HIC	Cross-sectional	survey	WwMS	192	100	/
Steinberg et al. (23)	Germany	HIC	Cross-sectional	mixed methods				
				survey	WwMS	95	100	/
				survey	WwMS	89	100	/
				interview	MS p	/	/	4
				focus group	WwMS	15	100	/
Soler et al. (45)	Chile	UMC	Retrospective cohort study	survey	WwMS	218	100	/
Wundes et al. (29)	USA	HIC	Cross-sectional	survey	MS p	/	/	28

Countries were categorized according to the World Bank income classification: High-Income Country (HIC), Upper Middle-Income Country (UMC), and Low- and Middle-Income Country (LMIC) (46). WwMS: women with multiple sclerosis. PwMS: people with multiple sclerosis. GP: general population, including WwMS and MS p. MS p: multiple sclerosis healthcare professionals.

### Key indicators

#### WwMS reproductive health

Overall, the most frequently explored topic across the included studies was “Impact of MS diagnosis on family planning and breastfeeding decisions”, examined in 22 out of 29 studies (76%). Within this category, 15 studies addressed how MS influences family planning choices, one assessed its effect on breastfeeding decisions, and 6 explored both. The second most common focus was “Counseling received by MS healthcare providers regarding reproductive health”, reported in 15 studies (52%), all of which assessed the content of information provided to patients.

Other reproductive health indicators were less commonly addressed. Lastly, “Age at first childbirth” was reported in 2 studies (7%), and “Rate of access to general prenatal care” was explored in only one study (18), which examined gynecological follow-up.

Notably, no study investigated the indicator “Completed family size for women currently aged 50-55”, leading to its exclusion from the final gap map.

#### MS healthcare professionals’ clinical practice

Several studies focused on how family planning guidance was delivered to WwMS. The most assessed topic was “Characteristics of the guidance provided to WwMS on family planning” (9/29 studies, 31%), with one study addressing the frequency of counseling, 2 (7%) evaluating its quality, and 6 (21%) addressing both issues. Clinical knowledge and use of recommendations appeared in four studies, one of which focused exclusively on contraception guidelines.

“Adherence to regulatory labels” was the least investigated, reported in a single study (19).

The key health indicator “Perceived self-efficacy about providing family planning counseling” was not identified in any study and has not been included in the gap map.

For a detailed overview, please see the linked interactive scoping map: ms.cochrane.org/sites/ms.cochrane.org/files/uploads/MAP_KNOWwMS.html [Accessed Dec 5, 2025].

## Discussion

This scoping review identified significant gaps in the available evidence about key information needs from the perspective of WwMS and health professionals engaged in their care.

A significant impact of MS diagnosis on reproductive decisions by WwMS about pregnancy and breastfeeding was the most reported issue across studies. Overall, the lack of familiar, social and professional support was widely reported, emphasizing the need for a structured approach that includes psychoeducational counseling, return-to-work planning, and tailored information programs for women with MS. (20) This evidence was complemented by the high number of studies that reported the characteristics of guidance provided by MS healthcare professionals, revealing several discrepancies both in the frequency and quality of the recommendations provided, with some providers reporting uncertainty about their role in addressing pregnancy, breastfeeding, and other family planning questions (7, 21).

The second most common issue was reproductive health counseling, with several studies reporting that this aspect was perceived as inadequate or inconsistent (9, 21–23). Women often reported initiating these discussions themselves (8, 24), and many described being discouraged from getting pregnant or breastfeed by professionals (9, 22, 23, 25, 26). Furthermore, many women continue to rely on the internet as their primary source of information (22, 27), a trend that might intensify in future years with the rise of online medical advice and AI tools (28).

Most studies were conducted in HICs, with a minority in UMICs. Notably, only one study (9) included in our review was conducted in a LMIC (Iran). This gap aligned with the findings of Ross et al. (10), who similarly noted the underrepresentation of limited resources setting perspectives in their review.

Considering the pre-identified key indicators, some of these were not prevalent within the literature. Indeed, only one study addressed the topic of “Rate of access to general prenatal care,” focusing on the gynecological care of WwMS, concluding that MS professionals should be more aware of the specific needs of these women and called for the development of international gynecological recommendations (18). Other studies in our review shared similar appeals for more guidance specifically tailored to WwMS of reproductive age (7, 24, 29). Moreover, only one study examined “Adherence to regulatory labels,” highlighting a potential gap in research assessing if existing recommendations and norms are actually being followed in clinical practice (19). However, practical indications on DMT use during childbearing years are available (6). We found lack of evidence about the perceived self-efficacy in providing family planning counseling by health professionals engaged in MS care. Indeed, none of the studies included in our review addressed this aspect, while its relevance in shaping the clinician-patient relationship has been reported in other settings (30, 31). Also, no study investigated completed family size in women over the age of 50, potentially missing an indicator.

Our review had several strengths, such as the comprehensive search of databases and the adoption of a rigorous recognized methodology during all stages of its development. Moreover, the structuring of review’s findings was informed by the SAG, that included representatives of two advocacy organizations of WwMS. Even though the focus of the review was WwMS, we also collected information on MS healthcare professionals, in order to deepen the understanding of where the potential gaps could derive from, considering both perspectives. The use of a gap map allowed for a structured and visual identification of the literature distribution.

This review also presents some limitations: firstly, the methods we followed to search and retrieve the evidence, although comprehensive, may not have covered all the evidence available on the topic of interest. Secondly, methods of collecting and presenting the data in the retrieved studies was heterogeneous, which may limit the interpretation of the findings. Finally, although the key indicators were advised by MS professionals and WwMS advocates, we did not seek or extract any additional outcomes that might have been reported in the included studies (e.g., reproductive health outcomes related to menstruation, menopause, etc.). Therefore, we may have missed further gaps in the evidence relative to other outcomes not mapped within our review.

A limitation of the available evidence emerging from this review is related to the prevailing designs of the included studies, most of which being cross-sectional, survey-based, with few using longitudinal designs or mixed methodologies. Survey-based studies may be particularly susceptible to recall bias, which could have affected the reliability of our findings. Likewise, the underrepresentation of studies from limited resources settings might have led to a skewed understanding of knowledge gaps and needs regarding reproductive health, reflecting primarily the experience of women living in HICs.

Further research should involve more longitudinal research with varied follow-up periods, that may better capture women’s informational needs across their lifespan. Mixed-method approaches could further enhance the depth and contextual relevance of findings, particularly in under-researched settings. Given the different challenges faced by healthcare systems in low-resources settings (32), more tailored research from LMICs could provide better information from WwMS living in these regions.

## Conclusion

The findings of our scoping review suggest that the available literature is mostly focused on assessing the impact of MS on family planning, with WwMS often being doubtful and insufficiently informed about key aspects of reproductive health. Addressing these knowledge gaps by means of a structured approach may not only help developing clear and valuable information for WwMS but also clarify the roles of MS healthcare professionals in counseling on pregnancy, breastfeeding and other aspects of family planning. Providing evidence-based and accessible information to WwMS and to health professionals is key for offering high-quality and equitable care, and to facilitate shared health decision-making in clinical practice. Furthermore, incorporating perspectives from LMICs and UMICs in future research could yield valuable insights for tailoring interventions to diverse cultural and resource contexts, ultimately enhancing reproductive health knowledge in limited resources settings.

## Data Availability

The raw data supporting the conclusions of this article will be made available by the authors, without undue reservation.
